# Metacognitive Therapy for Depression in Adults: A Waiting List Randomized Controlled Trial with Six Months Follow-Up

**DOI:** 10.3389/fpsyg.2017.00031

**Published:** 2017-01-24

**Authors:** Roger Hagen, Odin Hjemdal, Stian Solem, Leif Edward Ottesen Kennair, Hans M. Nordahl, Peter Fisher, Adrian Wells

**Affiliations:** ^1^Department of Psychology, Norwegian University of Science and TechnologyTrondheim, Norway; ^2^Institute of Psychology Health and Society, University of LiverpoolLiverpool, England; ^3^School of Psychological Sciences, University of ManchesterManchester, England

**Keywords:** metacognition, therapy, depression, treatment outcome

## Abstract

This randomized controlled trial examines the efficacy of metacognitive therapy (MCT) for depression. Thirty-nine patients with depression were randomly assigned to immediate MCT (10 sessions) or a 10-week wait list period (WL). The WL-group received 10 sessions of MCT after the waiting period. Two participants dropped out from WL and none dropped out of immediate MCT treatment. Participants receiving MCT improved significantly more than the WL group. Large controlled effect sizes were observed for both depressive (*d* = 2.51) and anxious symptoms (*d* = 1.92). Approximately 70–80% could be classified as recovered at post-treatment and 6 months follow-up following immediate MCT, whilst 5% of the WL patients recovered during the waiting period. The results suggest that MCT is a promising treatment for depression. Future controlled studies should compare MCT with other active treatments.

## Introduction

Depression has been described as one of the most common psychiatric disorders, with a high degree of comorbidity (Kessler et al., 2003). By 2030, depression is predicted to be the second-leading cause of disease burden worldwide after HIV/AIDS (Mathers and Loncar, 2006). It is therefore essential to develop effective treatments for depression. Cognitive-behavioral therapy (CBT) is the recommended treatment for depression, with a large number of clinical trials supporting its efficacy (Butler et al., 2006). However, only 40–58% of patients receiving CBT are recovered at post-treatment (Dimidjian et al., 2006). Relapse rates are between 40 to 60% within a period of 2 years (Hollon et al., 2006; Vittengl et al., 2007). Antidepressant medication has a similar efficacy in treating depression (Parker et al., 2008). It is therefore necessary to develop new treatments that have greater short-term and long-term efficacy.

A new treatment approach to depression that has produced encouraging results is metacognitive therapy (MCT; Wells, 2009). This approach is based on the metacognitive model where psychological disorder results from an inflexible and maladaptive response pattern to cognitive events labeled the Cognitive Attentional Syndrome (CAS; Wells, 2000; Wells and Matthews, 1994, 1996). The CAS consists of persistent worry and rumination, threat monitoring and ineffective coping strategies that contribute to the maintenance of emotional disorder. Rumination in depression is seen as a coping strategy which follows an initial negative thought labeled a ‘trigger thought’. The depressed individual engages in rumination consisting of repeatedly analyzing negative feelings, past failures and mistakes. Depression is therefore understood as an extension of low mood resulting from a problem of overthinking (e.g., worry and rumination) and withdrawal of active coping. (e.g., social withdrawal and reduction in activity). According to the metacognitive model of depression, rumination and worry is maintained by metacognitions and not by changes in mood or events. Further, this response to triggers extends negative thinking, leads to reduced attentional flexibility and involves a failure to exercise appropriate control over negative affective experiences (Wells, 2009).

According to the metacognitive model metacognitive beliefs control, monitor and appraise the CAS (Wells, 2009). There are both positive and negative metacognitive beliefs. Positive metacognitions are concerned with the benefits of worry and rumination, while negative metacognitions are concerned with the uncontrollability and danger of thoughts. Positive metacognitions related to depression may be exemplified by statements like: “Analyzing the causes of my sadness will give me an answer to the problem”, and “Thinking the worst will make me snap out of it”. Such positive metacognitive beliefs lead to repeated and/or prolonged engagement in ruminative thinking. Negative metacognitions are activated as the rumination process leads to distress and/or as a result of what the individual learns about depression. Examples of negative metacognitions are: “I can’t control my thinking”, “My thoughts are caused by my defective brain”, “Sleeping more will sort out my mind”. and “Thinking like this means I could have a mental breakdown”. Negative metacognitions lead to more distress and to unhelpful behaviors that reduce effective coping.

Metacognitive therapy aims to eliminate the CAS and to modify erroneous metacognitive beliefs to enable the development of greater flexible reactions to negative internal events. It does so by using behavioral experiments and verbal reattribution (Wells and Matthews, 1996; Wells, 2009), targeted at metacognitive change and specific techniques such as the attention training technique, detached mindfulness and postponement of rumination. According to metacogntive therapy this will enhance flexible executive control, and through the process of therapy the patient learns new and more beneficial ways of relating to thoughts that act as triggers for rumination (Wells, 2009). To clarify the differences between CBT and MCT; CBT focus on the content of thoughts and invites the patient to reality test this content, while in MCT thinking processes are addressed (for further descriptions of differences and similarities confer Fisher and Wells, 2009).

A recent meta-analysis of MCT for anxiety and depression concluded that MCT is effective and superior to waiting list and possibly CBT (Normann et al., 2014). The review by Normann et al. (2014) included two treatment studies on depression (Nordahl, 2009; Wells et al., 2012), one postpartum depression study (Bevan et al., 2013) as well as results from an unpublished study. Within-group effect size for depression trials in the review was 2.18 (Hedges *g*) at post-treatment. However, only the Nordahl (2009) study was a randomized trial and the primary problem was not exclusively depression. A previous study on MCT for depression was not included in the review: Wells et al. (2009) described MCT for four depressed patients of which three were recovered at 6 months follow-up. Recovery in the Wells study (2009) was defined using Frank et al.’s (1991) criteria, consisting of no longer having a diagnosis of depression and a Beck Depression Inventory (BDI) score of 8 or less. Since the publication of the review of Normann et al. (2014), several studies on MCT for depression have been published (Jordan et al., 2014; Callesen et al., 2015; Dammen et al., 2015; Papageorgiou and Wells, 2015). These studies also used Frank et al.’s (1991) criteria. The study by Callesen et al. (2015) described the treatment of four depressed patients of which three of them were recovered. The group-MCT study by Dammen et al. (2015) reported that 91% of the patients recovered at follow-up. Another group-MCT study (Papageorgiou and Wells, 2015) included 10 antidepressant and CBT resistant depression patients, and found that 70% were recovered at post-treatment and follow-up. The reported effect size reported with Hedge’s *g* was 2.88 at end treatment and 2.50 et 6 months’ follow-up. However, the small sample size of all these studies limits the generalizability of these results.

In the only controlled study of MCT in depression, 23 depressed patients were treated with MCT and compared with 25 patients treated with CBT (Jordan et al., 2014). Jordan et al. (2014) found that MCT and CBT produced similar positive results on symptom measures, but MCT produced superior effects on improved executive control (Groves et al., 2015). The reported effect sizes using Cohen’s *d* for intention to treat were 1.12 for MCT at end treatment. However, there were limitations in this study including low power, greater comorbidity in the MCT condition, and a lack of formal therapist training in MCT.

In summary, current recommended approaches for depression are CBT and antidepressant medication which produce moderate success rates and are often associated with significant relapse or recurrence. The metacognitive approach offers promising opportunities for addressing these limitations of treatment by directly targeting rumination and its underlying mechanisms that are seen as essential in the development and maintenance of depression (Wells, 2009). The present randomized controlled trial includes a larger sample of patients treated with MCT, and metacognitive therapist competency was ensured through training and supervision. We compared MCT with a waiting list control, since treatment studies do not take into account spontaneous remission in depression, as demonstrated by a mean decrease of 10–15% in depressive symptoms for waiting list control groups (Posternak and Miller, 2001). Furthermore, a WL condition can provide control over the effects of repeated assessment, regression to the mean and the expectancy of receiving treatment (e.g., optimism). Our prediction was that MCT would lead to greater improvement in depressive symptoms than a waiting period of 10 weeks.

## Materials and Methods

### Participants

The total sample consisted of thirty-nine participants and included 59% women (*n* = 23). The majority were ethnic Norwegians and three were Asian. The mean age was 33.7 years with a range from 18 to 54. Average number of children was 1.2. Three participants were currently treated with SSRIs. They were included as long as they agreed to maintain a stable dosage throughout the trial. A total of 30 (76.9%) participants had received earlier treatment for depression from their general practitioner, nine (23.1%) reported having been medicated with SSRIs for depression, 21 (53.8%) had received treatment from psychologists/psychiatrists at psychiatric outpatient clinics, three (7.7%) had previous inpatient treatment stays, and one had undergone ECT treatment.

A total of 16 participants were single, 15 were married or cohabitants, five had romantic partners, and three were divorced or separated. In all 12 patients worked full time, eight worked part time, seven were full time students, one was a part time student, while 13 were unemployed or received social or welfare benefits. With respect to education, two had completed elementary school, 17 had completed high school, five had 3 year college educations, and 15 had a 5 year university degree. Further demographic information on the sample is displayed in **Table 1**.

**Table 1:** Demographic and diagnostic information (*N* = 39).

	WL	MCT	Total pre	Total post
N	19	20	39	
	*M* (*SD*)	*M* (*SD*)	*M* (*SD*)	
Age	35.4 (8.8)	32.2 (11.7)	33.7 (10.4)	
	% (*n*)	% (*n*)	% (*n*)	
Women	52.6 (10)	65.0 (13)	59.0 (19)	
Norwegian ethnicity	100.0 (19)	85.0 (17)	92.3 (36)	
Married/cohabitant	42.2 (8)	35.0 (7)	38.5 (15)	
College/univ. degree	57.9 (11)	45.0 (9)	51.3 (20)	
Full time employed	42.1 (8)	20.0 (4)	30.8 (12)	
Part time employed	15.8 (3)	25.0 (5)	20.5 (8)	
Full time student	15.8 (3)	20.0 (4)	17.9 (7)	
Part time student	0.0 (0)	5.0 (1)	5.0 (1)	
Social/welfare benefits	26.3 (5)	40.0 (8)	33.3 (13)	
SSRIs current use	5.3 (1)	10.0 (2)	7.7 (3)	
SSRIs previous use	15.8 (3)	30.0 (6)	23.1 (9)	
GP treatment	84.2 (16)	70.0 (14)	76.9 (30)	
Psychiatric outpatient	63.2 (12)	45.0 (9)	53.8 (21)	
**Depressive episode**				
Mild	0	0	0	0
Moderate	1	2	3	1
Major	1	2	3	0
**Recurrent depression**				
Mild	1	0	1	3
Moderate	11	10	21	0
Major	5	6	11	0
**Axis I comorbidity**				
GAD	5	5	10	2
Social phobia	1	0	1	1
Hypochondriasis	1	0	1	0
Panic disorder	1	1	2	0
EDNOS	0	1	1	0
Binge eating disorder	1	0	1	0
Trichotillomania	0	1	1	1
*Total*	*28*	*28*	*56*	*8*
**Axis II comorbidity**				
Avoidant	1	2	3	1
OCPD	5	5	10	7
*Total*	*6*	*7*	*13*	*8*


*EDNOS = Eating Disorder Not Otherwise Specified, GAD = generalized anxiety disorder, Avoidant = avoidant personality disorder, OCPD = obsessive compulsive personality disorder, GP = general practitioner.*

Most of the sample had a recurrent depression diagnosis (79.5%) while 20.5% had a current depressive episode. The mean age at which the first depressive episode occurred was 26.2 years (*SD* = 11.7). The average duration of depression was *M* = 7.6 years (*SD* = 7.1). Comorbidity within the sample was as follows: 16 patients had one additional axis-I disorder (10 generalized anxiety disorder, two panic disorder, one social phobia, one hypochondriasis, one trichotillomania, and one eating disorder not otherwise specified), one patient also had a second comorbid axis-I disorder (binge-eating disorder). A total of 13 patients also had comorbid axis-II disorders (three avoidant personality and 10 obsessive compulsive personality disorders). Thirteen patients (33.3%) had depression as their single diagnosis.

### Procedure

This trial was registered at ClinicalTrials.gov (NCT01608399), and it was approved by the Regional Medical Ethics Committee in Norway (ref.nr. 2011/1138). Patients with primary depression disorder (mild, moderate, or severe) either single episode or recurrent depression were included (DSM-IV criteria). Further inclusion criteria for the study were signed written informed consent, and 18 years or older. Exclusion criteria were (a) known somatic diseases, (b) psychosis, (c) current suicide intent, (d) PTSD, (e) cluster A or cluster B personality disorder, (f) substance dependence, (g) not willing to accept random allocation, (h) patients not willing to withdraw use of benzodiazepines for a period of 4 weeks prior to entry to the trial, and (i) patients undergoing concurrent therapy elsewhere. A total of 105 diagnostic interviews were completed out of which 66 patients were excluded from the study. Reasons for exclusions were: GAD as primary diagnosis (*n* = 18), other primary diagnosis (*n* = 16), cluster A or B personality disorder (*n* = 10), no psychiatric diagnosis (*n* = 8), subclinical depression (*n* = 5), social phobia as primary diagnosis (*n* = 4), somatic diseases (*n* = 2), PTSD (*n* = 1), substance dependence (*n* = 1), and one patient was excluded due to use of anti-psychotic medication.

Participants were recruited between January 2013 and January 2015. Participants were treatment-seeking individuals referred by their GP or self-referred to a university outpatient clinic in a major city in Norway. Information about the study and descriptions of how to refer participants were provided in local newspapers, as letters to GPs, on the radio and through advertisements on social media.

After initial telephone screenings, potential participants met with a trained assessor who provided detailed information about the study, obtained informed consent and reviewed inclusion and exclusion criteria and severity of depression and other psychiatric conditions. The diagnostic interviews included Structured Clinical Interview for the DSM IV axis I (SCID-I; First et al., 2002), Structured Clinical Interview for the DSM IV axis II (SCID-II, First et al., 1997), and the Hamilton Rating Scale for Depression (HRSD-17; Hamilton, 1967). The assessment team conducted the interviews at pre- and post-treatment, while post-waiting list and follow-up data was based on self-report. Agreement upon diagnosis was achieved by conferring with two senior researchers who watched videotaped recordings of the interviews. Assessments were completed before treatment, after the wait period (waiting list group only), after treatment, and at 6 months follow-up. Consenting participants who met inclusion criteria were randomly assigned to either begin MCT immediately or after a 10-week wait period.

**FIGURE 1:**
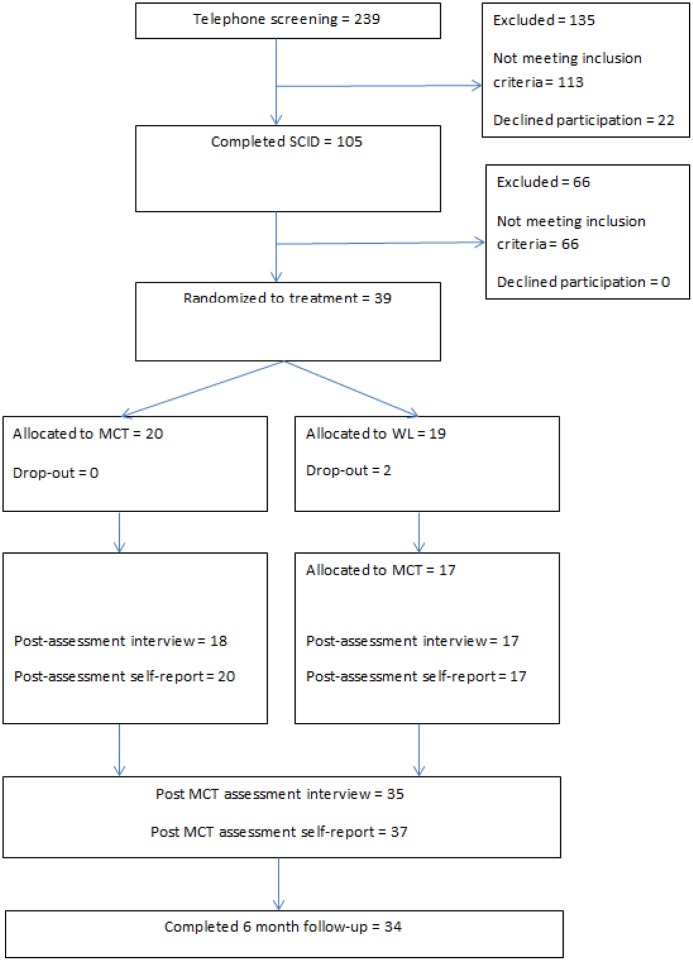
**Flow chart**.

Sample size was calculated based on a minimum clinically meaningful difference between treatments on BDI of 7. Accepting a probability of Type I error of 5 per cent with 80 percent power (Chandrashekara, 2012), 17 patients would be required in each group. Estimating with an expected attrition rate of 30% would indicate group sizes of 22. Given that the attrition rate was low, inclusion was stopped at 39 patients. A randomization schedule was generated by Excel’s random number generator prior to the project start. Two factors were controlled for in the randomization; gender and recurrent depressive episodes. **Figure 1** illustrates participant flow through the study.

### Measures

#### Structured Clinical Interviews

Structured clinical interviews were performed both at pre- and at post-treatment with the SCID-I and SCID-II and theHRSD-17. Diagnoses were assessed using the SCID-I and SCID-II. The patients were re-assessed with SCID-I and SCID-II at post-treatment using only the modules corresponding to their pre-treatment diagnoses. This was to assess if they had the same diagnosis after having undergone treatment. The HRSD-17 is a structured interview that includes items related to depressive symptoms, 0–4 scale, where 0 correspond to the absence of symptoms, and the score of 4 corresponds to severe symptoms. Scores between 0 and 6 do not indicate the presence of depression, scores between 7 and 17 indicate mild depression, scores between 18 and 24 indicate moderate depression, and scores over 24 indicate severe depression.

#### Self-Report Instruments

The Beck Depression Inventory (BDI; Beck et al., 1961) is a 21-item self-report inventory used to measure level of depressive symptoms. Each item is rated on a four-point Likert-type scale ranging from 0 to 3, indicating the severity of each symptom. The BDI has been extensively shown to be a reliable and valid measure of severity of depressive symptoms in both clinical- and non-clinical populations (Beck et al., 1988). Beck and colleagues categorized the BDI total scores as follows: 0–9 minimal, 10–18 mild, 19–29 moderate, and 30–63 severe depression.

The Beck Anxiety Inventory (BAI; Beck and Steer, 1990) is a 21-item self-report inventory for assessing the presence and severity of anxiety symptoms. Each item is rated on a four-point Likert-type scale ranging from 0 to 3, indicating the severity of each symptom. Beck and Steer (1990) categorized the BAI total scores as follows: 0–7 minimal, 8–15 mild, 16–25 moderate, and 26–63 severe. For psychometric properties of the BAI, see Steer et al. (1993).

### Treatment

Patients received 10 sessions of MCT for depression following the published treatment manual and session guides for depression (Wells, 2009). Briefly, the treatment consisted of: case conceptualization and socialization which are undertaken first and then followed by (1) increasing meta-awareness by identifying thoughts that act as triggers for rumination, learning about metacognitive control using attention training; (2) challenging beliefs about the uncontrollability of rumination and worry; (3) challenging beliefs about threat monitoring and dangers of rumination and worry; (4) modification of positive beliefs about rumination and worry; and (5) relapse prevention.

### Therapists

Therapists were clinical psychologists who had all received previous training in MCT. Treatment was supervised by professor Adrian Wells (AW), the originator of MCT, to ensure quality of the delivered treatment. AW watched videotaped recordings of sessions and provided ongoing feedback. The tapes were simultaneously translated by the bilingual therapists. The therapists met every month for peer supervision. No formal measure of therapists’ competence, treatment integrity or adherence was obtained. A split plot ANOVA found no significant differences between therapists with respect to changes in HRSD scores, *F*(3,34) = 0.942, *p* = 0.43).

### Data Analyses

A mixed-model repeated ANOVA was used to compare the MCT group with the waiting list control. *Partial eta* squared statistics and Cohen’s *d* effect sizes (using pooled standard deviations) were used to estimate the treatment effects and between group differences. A repeated measures ANOVA and *t*-tests was also used to estimate the treatment effects for the total combined sample (those who received immediate and delayed treatment), entering BDI and BAI scores from pre-treatment, post-treatment, and 6 months follow-up. Controlled effect sizes were calculated in the following way: post treatment for immediate MCT minus post waiting list divided by the pooled standard deviation. To evaluate clinically significant outcomes, Jacobson criteria (Jacobson et al., 1999) was used, with a cut-off point (14) and reliable change index (8.46, which was rounded up to 9 in the present study) obtained for the BDI (Seggar et al., 2002). Both ITT and completer analyses are reported on outcome measures and clinically significant change analyses, thus allowing for comparison with existing studies as recommended (Hiller et al., 2012).

For the ITT analysis we used last observation carried forward to replace missing values. The two dropouts from the waiting list condition were missing BDI and BAI scores at post-treatment and follow-up. They were assigned their pre-treatment scores at post-treatment and follow-up. There was very little missing data on individual BDI items (0.4%) and BAI items (0.8%). In these cases missing items were replaced using mean item scores on the remaining items.

All participants allocated to receive MCT immediately after randomization completed treatment. Two participants allocated to waiting list dropped out during the waiting period (one moved and one started treatment at a private practice psychologist) and did not provide data after pre-treatment. These two were included in the intent to treat analyses and their post-treatment results were replaced using last observation carried forward. Of the patients initially allocated to waitlist and then going on to delayed treatment, two of them did not go on to complete all 10 treatment sessions. These two patients did not meet with the assessment team for a post-treatment and follow-up interview, however self-report data was available from their latest treatment sessions and used as post-treatment results. Thus, a total of 17 patients completed MCT after first being allocated to waiting list resulting in a total of 35 post-treatment interviews. All except one of these 35 also completed self-report questionnaires at 6 month follow-up.

## Results

**Table 1** presents an overview of demographic and diagnostic information for the two groups and the total sample. The two groups were similar on all variables with no statistically significant differences between them at pre-treatment. With respect to diagnoses there were a total of 57 axis–I diagnoses given at pre-treatment for all the participants. This number was reduced to 8 axis-I at post-treatment. Four patients still had a diagnosis of depression (3 with mild recurrent depression and 1 with moderate depressive episode). With respect to comorbid conditions, 3 patients still suffered from GAD, one from social phobia and one from trichotillomania. As for axis-II comorbidity, there were 13 diagnoses given at pre-treatment and 8 at post-treatment, suggesting that 40% also recovered from their personality disorder as assessed by SCID-II.

### Primary Outcomes

#### BDI

To assess the comparative outcomes a mixed model ANOVA was run on the BDI measured at pre and post treatment. **Table 2** shows the main effect of time *F*(1,37) = 140.59, *p* < 0.001 and the group by time interaction, which was significant *F*(1,37) = 80.05, *p* < 0.001. Inspection of the group means indicated that the MCT group showed greater improvement in BDI scores than the wait list group from pre to post. The within subject contrasts on a group-wise basis confirmed that both the MCT and the wait list group improved from pre to post intervention. The within group effect sizes (*d*) were; MCT = 3.50, WL = 0.49, with a controlled effect size of *d* = 2.51.

**Table 2:** Descriptives and Mixed-model ANOVA results for depression and anxiety symptoms and paired sample *t*-test for within group comparison for MCT and waiting list, respectively.

	Pre			Post		Between MCT-WL			Within-group	
				
	*n*	*M*	*SD*	*M*	*SD*	*F*	$\eta_{p}^{2}$	*d*	*t*	*d*
**BDI**										
MCT	20	27.85	6.85	6.40	6.84	140.59^∗∗∗^	0.79	2.51	12.68^∗∗∗^	3.50
WL	19	26.89	5.61	23.89	7.05				2.63^∗^	0.47
**BAI**										
MCT	20	22.60	10.25	3.65	7.70	45.06^∗∗∗^	0.55	1.92	8.03^∗∗∗^	2.09
WL	19	19.16	7.97	18.42	7.71				0.44	0.09


*BDI = Beck Depression Inventory; BAI = Beck Anxiety Inventory; MCT = Metacognitive therapy; WL = Wait-list. HRSD-17 was not administered at post waiting-list. ^∗^*p* < 0.05, ^∗∗∗^*p* < 0.001.*

#### HRSD

In the wait list group the HRSD was only assessed prior to waiting and after the provision of delayed treatment. Therefore, comparison between the waitlist group and MCT is not possible. We ran a repeated measures *t*-test for the whole sample to assess overall change in HRSD associated with treatment (ITT analysis, see **Table 3**). For the entire combined sample the ITT showed larger effect size of *d* = 2.95.

**Table 3:** Means, standard deviations and effect sizes at pre-treatment, post-treatment and 6 months follow-up for the MCT group and the total combined sample with repeated sample *t*-tests for the Hamilton Rating Scale for Depression and repeated measures ANOVA and repeated sample *t*-tests for BDI and BAI.

	Pre-treatment		Post-treatment				Follow-up			
			
	*M*	*SD*	*M*	*SD*	*t (pre-post)*	*d*	*M*	*SD*	*t (post-FU)*	*F*
HRSD-17										
MCT ITT	19.65	3.42	5.35	7.41	8.45^∗∗∗^	2.53				
All ITT	19.92	3.58	5.33	6.54	14.59^∗∗∗^	2.95				
All Completers	20.29	3.66	3.56	4.87	16.74^∗∗∗^	4.81				
BDI										
MCT ITT	27.85	6.85	6.40	6.84	12.68^∗∗∗^	3.13	7.55	8.96	–0.90	76.20^∗∗∗^
All ITT	25.92	7.14	6.64	8.03	19.28^∗∗∗^	2.53	8.21	9.45	–1.74	162.34^∗∗∗^
All Completers	25.69	7.36	4.77	5.75	20.91^∗∗∗^	3.45	6.51	8.19	–1.75	172.75^∗∗∗^
BAI										
MCT ITT	22.60	10.25	3.65	7.78	8.025^∗∗∗^	2.08	6.75	10.84	–1.98	30.90^∗∗∗^
All ITT	20.56	9.22	4.84	7.22	15.72^∗∗∗^	1.98	7.00	9.56	–1.77	97.71^∗∗∗^
All Completers	20.83	9.33	3.60	5.01	17.23^∗∗∗^	2.34	6.00	8.64	–1.77	104.47^∗∗∗^


*HRSD-17 = Hamilton Rating Scale for Depression; BDI = Beck Depression Inventory; BAI = Beck Anxiety Inventory; ITT = Intention To Treat. ^∗∗∗^*p* < 0.001.*

### Secondary Outcomes

#### BAI

A mixed model ANOVA was run to assess the comparative outcomes on the BAI measured at pre and post-treatment. **Table 2** presents the main effects of time *F*(1,37) = 45.06, *p* < 0.001 and the significant group by time interaction *F*(1,37) = 38.57, *p* < 0.001. The group means indicated that the MCT group showed greater improvement in the BAI scores than the wait list group from pre to post. The within subject contrasts on a group-wise basis confirmed that both the MCT and the wait list group improved from pre to post intervention. The within group effect size (*d*) were; MCT = 2.09, WL = 0.09, with a controlled effect size of *d* = 1.92.

### Follow-Up Results

To assess the stability of the treatment effects repeated measures *t*-tests for BDI and BAI were used to determine any significant changes from post treatment to 6 months follow-up assessments. There were no significant changes within this time period for any of the measures (see **Table 3**). Very large effect sizes were observed on the HRSD-17, BDI and BAI from pre to post treatment. The large effect sizes were somewhat lower from pre to 6 months follow-up with BDI of 2.11 and BAI of 1.44 for the intention to treat sample (combined groups).

#### Combined Sample

To provide more accurate estimates of effects for the purposes of future sample size estimates the analyses on the combined sample of patients from pre to post treatment on HRSD-17 and pre, post, and follow-up on BDI and BAI were calculated. In **Table 3** the within subjects t-tests showed significant improvements on HRSD, BDI, and BAI, with high effect sizes ranging from 1.98 to 3.13 for the intention to treat sample and from 2.34 to 4.81 for the treatment completers.

### Clinically Significant Change Analyses

**Table 4** presents an overview of clinical significant change for patients that did not change, improved, or were classified as recovered after treatment. None deteriorated at post treatment or follow-up. For the HRSD-17, the immediate MCT intention to treat effects showed 75% were classified as recovered, while for the entire group with intention to treat more than 82.4% were classified as recovered. According to the criterion set by Jacobson and colleagues (1999), the BDI results were similar and indicated that approximately 80% of the MCT intention to treat sample was recovered at post-treatment and 75% at 6 months follow-up. For the entire group in the intention to treat condition over 79.5% were recovered at post treatment and over 69.2% at 6 months follow-up. In the waiting list condition 2.6% recovered, and 2,6 % were improved during the 10 weeks of waiting. No patients deteriorated following treatment. No harm or unintended effects were reported or observed. There were no significant differences for moderate and severe recurrent depression, neither from pre to post χ^2^(1) = 0.13, *p* = 0.72 nor from pre to follow-up χ^2^(1) = 0.03, *p* = 0.86 on BDI. There were too few participants with episodes or mild recurrent depression to include these in the analyses.

**Table 4:** Clinically significant change in depressive symptoms for the MCT immediate treatment group (*n* = 20) and the total combined sample (*N* = 39).

	Pre-post				Pre to F–U			
		
	N	No change	Improved	Recovered	*N*	No change	Improved	Recovered
HRSD-17								
MCT ITT	20	25.0%	–	75.0%				
All ITT	39	28.2%	–	71.8%				
All Completers	34	17.6%	–	82.4%				
BDI								
MCT ITT	20	5.0%	15.0%	80.0%	20	10.0%	15.0%	75.0%
All ITT	39	7.7%	12.8%	79.5%	39	12.8%	17.9%	69.2%
All Completers	37	2.7%	13.5%	83.8%	35	5.7%	17.1%	77.1%


*MCT ITT = MCT immediate group only with intention to treat; All = the total sample; ITT = Intention to treat; HRSD-17 = Hamilton rating scale for depression; BDI = Beck Depression Inventory. No patient deteriorated. HRSD-17 criteria for recovery = scores from 7 and below. BDI criteria for improvement = patients that had a 9 point reduction on the BDI or BDI of 14 or less. BDI criteria for recovery = patients that had a 9 point reduction on the BDI and a score lower than 14 points.*

### Return to Work Outcomes

At post-treatment 30.6% had started working or studying, 58.3% were still in work/studies, and 11.1% were still unemployed or on disability benefits. Unemployment or disability benefits were reduced by 22.2% from pre to post-treatment. Three of the patients were unaccounted for with respect to employment status.

## Discussion

The results of this trial of MCT for depression are encouraging with large and statistically significant reductions both for depressive and anxious symptoms following 10 sessions of treatment. These improvements were sustained at 6 months follow-up. The clinical significance analyses showed that 70–80% of the total sample of patients achieved recovery which is consistent with previous uncontrolled studies on MCT. These numbers suggest that the majority of patients benefitted from MCT and none of the patients reported a worsening of symptoms. The comparison of the MCT group with the control group allows us to partial out the effects of time and factors such as expectancy from ‘true’ treatment related effects. The effect size for the control group was moderate suggesting that these effects are by no means insubstantial. However, MCT produced a much greater effect supporting attribution of the outcome to aspects of the intervention itself.

Several meta-analyses have examined the effect of CBT for adult depression. A meta-analysis containing 78 studies reported that the comparison between CBT and waiting-list had an effect size of 0.82 on the BDI (Gloaguen et al., 1998). An updated meta-analysis by Cuijpers et al. (2013) shows similar effect sizes. In their review 115 studies met the criteria for inclusion, and the mean effect size related to CBT vs. control groups was Hedges *g* = 0.83. The analysis suggests the effect-size to be overestimated caused by publication bias and poor quality of some studies included in the meta-analysis. The present study comparing MCT and waiting-list found an effect size of 2.53 on the BDI. There are large differences between the effect sizes previously reported for CBT and presently reported for MCT, whilst these differences suggest advantages for MCT they may be biased by the small number of studies on MCT and allegiance effects. These findings emphasize the need for future studies with a direct comparison of CBT with MCT in larger studies.

Regarding personality disorders, the study found that five patients out of 13 did not meet criteria for personality disorder at post-treatment. Other studies on metacognitive therapy have also shown promising results despite the presence of personality disorders (Wells et al., 2012; Dammen et al., 2015; Hjemdal et al., 2016), but also that characterological symptoms could change following MCT. In fact, Dammen et al. (2015) reported that all five patients with personality disorders no longer met criteria at post-treatment. The loss of such diagnoses following MCT for depression might imply that the features were a manifestation of their cognitive attentional syndrome and that modifying this system and its underlying metacognitions could involve changes in self-perceptions and habits. However, these results also mirror findings showing that change in depression could in general co-occur with change in personality (e.g., Fava et al., 2002). This could also reflect that personality disorder diagnoses might be confounded by affective states of the patients. The prominent symptoms in avoidant- and obsessive compulsive personality disorders are avoidance, low self-esteem, perfectionism, and inflexibility. Future research should investigate whether these changes in personality are maintained at long-term follow-up or merely a consequence of changes in depressive symptoms. Some consideration should be given to the representativeness of the current sample Only one in four had used or were using anti-depressive medication in the study. This is, however, comparable to other Norwegian studies of psychotherapy interventions for depression (Dalgard, 2006; Høifødt et al., 2013; Dammen et al., 2015). Furthermore, half of the sample had been in previous psychiatric outpatient treatment. Taken together these observations suggest that the sample could be representative of a depressed treatment-seeking patient group.

A potential limitation concerns the fact that no assessment of treatment adherence and therapist competence were performed. Whilst adherence to treatment was monitored throughout supervision there was no formal assessment of adherence to the treatment manual. There were no differences between therapists with respect to patient changes in depressive symptoms, which may suggest that therapist differences did not affect the results significantly. Questions might be raised related to the design using a waiting list as a control condition. Although Furukawa et al. (2014) found that waiting list could have a nocebo effect, the knowledge of the course of untreated depression can serve as a benchmark for assessing the true benefits of active treatment. In the short-term, depressive symptomatology can be expected to decrease by about 10–15% on average without treatment (Posternak and Miller, 2001). The sample size may imply some limitations regarding internal and external validity of the study. Another potential limitation concerns the fact that missing data was handled using last observation carried forward. This can be problematic when it reduces effects such as regression to the mean which would become a problem if there was greater missing data in one condition compared with the other. In the present study, the extent of data missing was small and therefore it is likely to be a conservative method of replacing missing values.

Further, the inter-rater reliability for the HRSD-17 is lacking. Moreover, the assessors were not blind to involvement in treatment or the hypothesis of the study, but they were however blind to group assignment at pre-treatment. Future studies should investigate long term efficacy of MCT for depression and the relationship between changes in rumination and metacognitive beliefs and symptoms. Finally, randomized controlled trials with active treatment comparisons are essential before we can be more confident of the effects of this treatment.

## Conclusion

Metacognitive therapy was associated with large improvements in depressive symptoms. Comorbid disorders and symptoms were also improved. Treatment gains were large and sustained for 6 months follow-up. There were no significant differences in treatment effect for patients with moderate- or severe recurrent depression. These results support MCT as a potentially effective treatment for depression that could lead to improved outcomes.

## Ethics Statement

All subjects gave written informed consent in accordance with the Declaration of Helsinki. The protocol was approved by Regional Medical Ethics Committee in Norway (ref.nr. 2011/1138).

## Author Contributions

RH, OH, SS, LK conducted the therapy in the trial. All authors have contributed equally in writing up the manuscript.

## Conflict of Interest Statement

The authors declare that the research was conducted in the absence of any commercial or financial relationships that could be construed as a potential conflict of interest.
